# “Gauze Technique” in the Treatment of the Fungus Ball of the Maxillary Sinus: A Technique as Simple as It Is Effective

**DOI:** 10.1155/2016/4169523

**Published:** 2016-12-19

**Authors:** Pietro Garofalo, Alessandro Griffa, Georges Dumas, Flavio Perottino

**Affiliations:** ^1^ENT Department, University of Piemonte Orientale, Novara, Italy; ^2^ENT Department, Centre Hospitalier “Les Escartons”, Briançon, France

## Abstract

Fungus ball of maxillary sinus generally affects immunocompetent and nonatopic subjects. Although endoscopic removal is the current gold standard treatment, removal is at times difficult due to an accumulation of fungal elements in the anterior ad inferior recesses.* Aim*. To present our experience of maxillary fungus ball treated by the “gauze technique” that avoids these removal difficulties.* Materials and Methods*. A retrospective, cross-sectional, and descriptive study of 25 patients affected by maxillary fungus ball was carried out: 19 were treated by the “gauze technique” and 6 were treated without “gauze technique.”* Results*. A comparison was made between the two groups for surgery procedure time, length of hospitalization, time from surgery to nasal unpacking, complications, and postsurgical patient satisfaction. The only statistically significant difference observed was a shorter surgical procedure time (*p* < 0.05) for the “gauze technique.”* Conclusions*. The data obtained in this study demonstrated that the “gauze technique” is a safe, simple, and quick technique, able to reduce surgery procedure time whilst providing excellent functional outcomes and patient satisfaction.

## 1. Introduction

The term fungus ball (FB) refers to a noninvasive mycosis of the paranasal sinuses that affects immunocompetent hosts and frequently affects one single sinus. Although fungi are normal saprophytes of the nasal cavities and paranasal sinuses, under particular conditions, they may lead to specific sinonasal diseases. These conditions are favoured by hypoxia and low intrasinusal pH values due to ventilation disturbance and impairment of mucociliary clearance, where the closure of the natural ostium of paranasal sinus is the underlying pathogenesis [1].

Paranasal fungal sinusitis have been classified into two categories, according to DeShazo classification (1998) which takes into consideration the presence/absence of sinonasal mucosa invasion: the noninvasive forms and the invasive forms [2]. The former include the sinus fungus ball (once called mycetoma or aspergilloma) and allergic fungal rhinosinusitis which typically affect immunocompetent subjects. The latter include acute fulminant rhinosinusitis, chronic invasive fungal rhinosinusitis, and granulomatous fungal rhinosinusitis, which typically affect immunodeficient subjects [3]. As aforementioned, FB generally affects immunocompetent subjects and is an extramucosal fungal proliferation, characterized by a mass of inspissated fungal debris and mucus that grows progressively in the sinus cavity, completely filling one or more of the paranasal sinuses, sparing the underlying mucosa [4]. Colonisation is mostly caused by* Aspergillus* spp. and has been found in the maxillary or sphenoid sinus in more than 80% of FB patients, whilst ethmoidal, frontal, or multiple localizations are rarer [3, 4].

**Figure 1:**
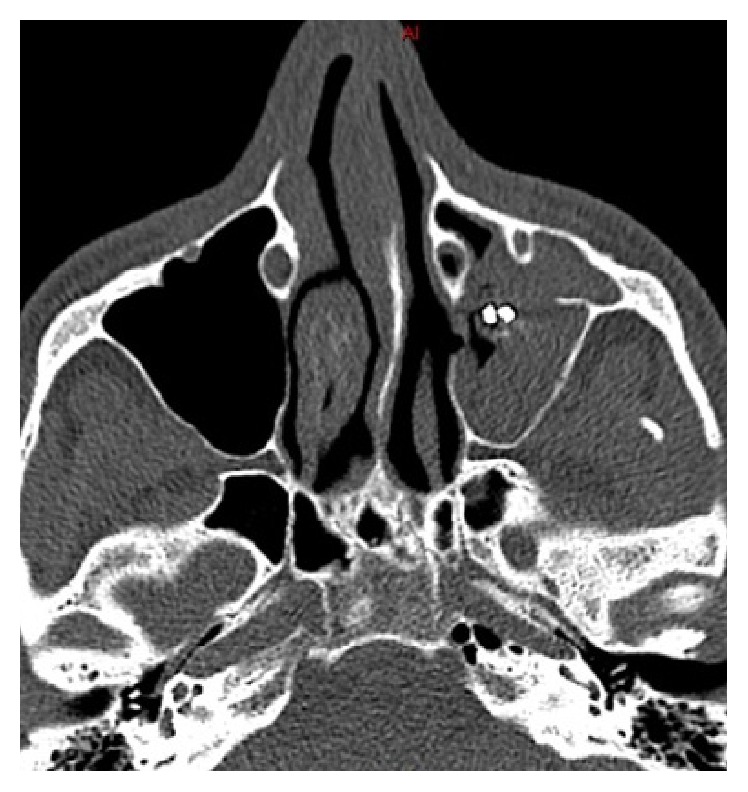
CT facial scan that shows the aspect of an endosinusal foreign body.

The fungus ball is typically asymptomatic in the early stages and signs and symptoms may take several years to appear. Moreover, its clinical manifestation is often nonspecific and variable and includes nasal congestion, purulent or blood-stained nasal discharge, headache, craniofacial pain, and/or an impaired sense of smell [5]. Radiographic assessment is to be started with a computed tomography (CT) scan without contrast medium. The disease has a distinct radiographic appearance, which includes hyperdense focal areas and/or the presence of an endosinusal foreign bodies or “iron-like” bodies (Figure 1). If there remains doubt, T2-weighted magnetic resonance imaging (MRI) could be useful, where calcifications and paramagnetic metals [1] generate areas of signal void in correspondence with the diseased tissue [1, 6].

The mainstay of treatment for FBs is the surgical excision aimed at improving sinus aeration and minimising mucosal loss. Endonasal removal by a purely endoscopic approach is unanimously considered the treatment of choice and has provided excellent results [7, 8]. However, this disease commonly involves the anterior recess of the maxillary sinus making its removal with the endoscopic technique both harder and longer due to the difficulty to reach this maxillary region. Herein, we report a simple and timesaving technique, proposed by Chao and Liu in 2006, able to obviate this problem [9]. It involves the use of traditional endoscopic instruments, together with standard gauze used to “clean out” the FB from the maxillary sinus without resorting to any destructive procedures. It is a safe technique that does not present more complications than those reported for the classical endoscopic technique [9].

## 2. Materials and Methods

A retrospective study was carried out on data from a review of the records of 66 patients operated on by the same surgeon, from 2006 to 2014, at the Otorhinolaryngoiatric Unit of the “Centre Hospitalier des Escartons” of Briançon, France, with antrostomy for unilateral maxillary sinusitis and suspicion of a fungus ball. Inclusion criteria were patients who had been given a definitive anatomopathological and/or histological diagnosis of fungus ball of maxillary sinus, patients without conditions at diagnosis that could affect the development of sinonasal fungal disease (such as immunodeficiency, allergy, or previous endoscopic nasal surgery), and patients who had been operated on by the same chosen surgeon. The study group included 25 patients who met the inclusion criteria. The study group was divided into two groups and endoscopic removal of the maxillary FB was performed with the gauze technique in 19 cases and without the gauze technique in 6. Bacteriological and histological confirmation of a fungus ball of the maxillary sinus was obtained in all cases.

The study variables were the general characteristics of the patients (age, gender, comorbidities, and symptoms), the surgical procedures (duration in minutes, the use of the gauze technique, and any complications), length of hospitalization, time-lapse to nasal unpacking, duration of the follow-up, postoperatory events and evaluation of objective outcomes, and postsurgical patient satisfaction, which was evaluated by the SNOT-20 (20-Item Sinonasal Outcome Test) [10].

Surgery comprised 2 groups: the group treated with gauze technique and the group treated without gauze technique. We estimated medians and ranges for continuous variable and percentage for categorical variables. The differences between the gauze technique and the classic technique were evaluated in terms of age, the length of the surgical procedure, time to nasal unpacking, negative postoperative events, or complications and the surgical outcome was evaluated by the SNOT-20.

The results between the two groups were compared by the Mann–Whitney *U* test and Fisher exact test. All *p* values are 2-tailed and the statistical significance cut-off was *p* < 0.05.

### 2.1. Surgical Technique

A wide antrostomy was made following the classical endoscopic technique. The bulla ethmoidalis was identified after medial luxation of the median turbinate. A curved aspirator was then used so as to palpate under the bulla, in the so-called “fontanelle area,” a thin portion of osteomeatal complex, so as to identify the sinus. The antrostomy was then enlarged with retrograde pincers in a posterior versus anterior direction. The fungus ball was then visible through the antrostomy and was first removed with the curved endoscopic pincers (Blakesley-Weil 45° and 90°) and suction.

**Figure 2:**
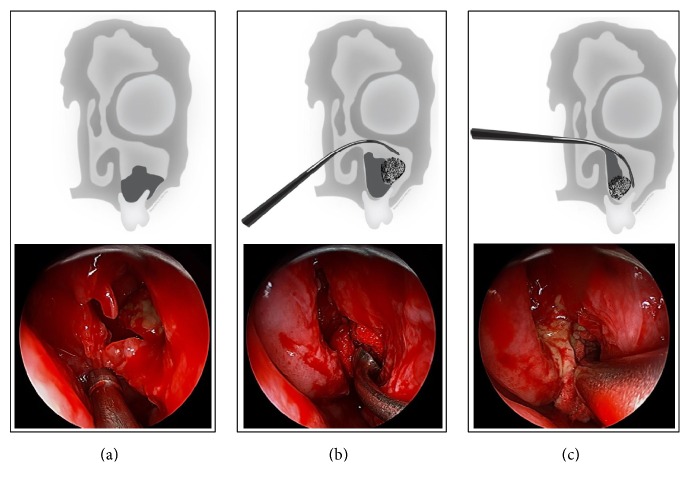
Important phases of intervention: (a) wide antrostomy; (b) introduction of the gauze in the maxillary sinus; (c) pushing the fungal material towards the middle meatus.

In the 19 cases operated on with the gauze technique, a 5 × 5 cm piece of gauze was divided into two strips. One of the strips was soaked in standard saline solution and stretched to form a long strip. This was then introduced into the nasal cavity and through the antrostomy into the maxillary sinus. The bulk of the gauze then pushed out the remaining fungal elements; a curved suction tube and curved pincers, the so-called “J-curette” for maxillary sinus, pushed the gauze laterally and inferiorly, remaining in contact with the anterior wall of the maxillary sinus, to allow the fungal material to move out towards the maxillary antrostomy. At this point, the residual fungus ball was easily removable through the middle meatus. Noteworthy is the fact that when the gauze is removed through the nostril, it often has FB fragments attached. The surgical procedure is schematized in Figure 2. The maxillary sinus was then explored with a 45° and 70° endoscope so as to identify any residual fungi, which, if present, are usually located in the anterior maxillary recess.

**Figure 3:**
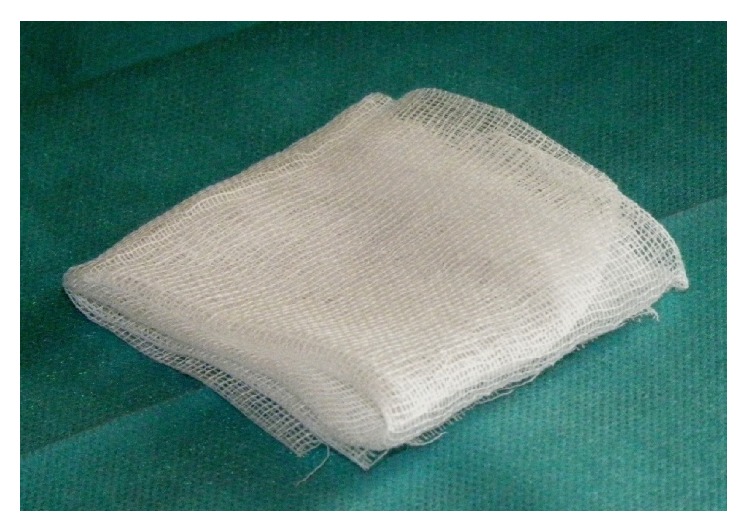
Braided-mesh gauze.

The same procedure can be repeated several times, after which the gauze can be easily removed with curved forceps. The use of a braided-mesh gauze is a must (Figure 3), to enable a thorough debridement of all the pathological material. The maxillary sinus was washed at the end of the procedure, firstly with a diluted antifungal solution and then with a saline and peroxide solution. Every patient was discharged after nasal unpacking with a therapy that included oral antibiotic (amoxicillin-clavulanate for 5 days), oral analgesics (paracetamol for 10 days), and nasal washes based on saline solutions for 1 month.

## 3. Results

A total of 25 patients were given a diagnosis of fungus ball of the maxillary sinus, from 2006 to 2014, at the Otorhinolaryngologic Unit of the “Centre Hospitalier des Escartons” of Briançon; eleven were male and fourteen female.

The median age was 55.0 years, ranging from 28 to 87. The symptoms presented by patients included nasal congestion and impaired sense of smell in 21 patients (84.0%), purulent nasal discharge at least once in 18 patients (72.0%), and craniofacial pain in 11 (44.0%) patients.

Diagnosis workup included a facial CT scan without contrast: in 23 patients (92.0%), areas of hyperdensity in the central portion of the affected maxillary sinus and/or image of calcifications (mimicking a foreign metal body) were observed. Two patients (8.0%) had aspecific signs of sinusitis. Of 25 patients diagnosed with fungus ball of maxillary sinus from 2006 to 2014 at the Otorhinolaryngology Unit of the “Centre Hospitalier des Escartons” of Briançon, eleven were male and fourteen were female. The median age was 55.0 years ranging from 28 to 87 years. None of the patients had conditions at diagnosis that could affect the development of sinonasal fungal disease (such as immunodeficiency, allergy, or previous endoscopic nasal surgery).

Symptoms presented by patients included nasal congestion and impaired sense of smell in 21 patients (84.0%), purulent nasal discharge at least once in 18 patients (72.0%), and craniofacial pain in 11 (44.0%) patients.

Diagnosis workup included for every patient a facial CT scan without contrast: in 23 patients (92.0%), we found areas of hyperdensity in the central portion of the affected maxillary sinus and/or image of calcifications (miming a foreign metal body). In 2 patients (8.0%), we found aspecific signs of sinusitis.

The gauze technique intervention was adopted to treat maxillary fungus ball from 2010, and among 25 patients, 6 were treated without gauze technique whilst 19 were treated with gauze technique. The median age at surgery was 54.0 years, ranging from 24 to 83 years. The median surgical procedure time was 70.0 minutes, ranging from 30 to 180 minutes. All patients had postsurgical nasal packing. One of them (4.0%) was unpacked 6 hours after surgery, 16 (64.0%) were unpacked 24 hours after surgery, and 8 (32.0%) were unpacked 48 hours after surgery. The timing of the unpacking depended on the entity of the bleeding during surgery.

The median hospitalization length was 3.0 days: 2 patients (8.0%) were hospitalized for 1 day, 5 (25.0%) for 2 days, 9 (36.0%) for 3 days, 5 (20.0%) for 4 days, and 4 (16.0%) for 5 days. There was only one postsurgical complication (4.0%), an epistaxis on the 5th postsurgical day, which was treated successfully by nasal packing for 48 hours. One other patient had a relapse at one year, treated with Caldwell-Luc approach with success. The follow-up ranged from 1 to 105 months, for a median of 46.0 months. Positive follow-up outcome data were high. Indeed, the median SNOT-20 score was 3.0, ranging from 0 to 35, where 0 is the absence of symptoms and 100 is the highest number of symptoms ever recorded.

Table 1 reports the data from the clinical notes of the 25 patients.

Table 2 reports the comparison between the gauze technique and the classical-technique in terms of age, surgery timing, hospitalization length, SNOT-20 results, timing of unpacking, and number of postoperatory complications. The only statistically significant difference observed between the two groups was a statistically significantly lower surgical procedure timing for the gauze technique (*p* < 0.05).

## 4. Discussion

Surgery is the treatment of choice for fungus ball which has the role of removing fungal debris from the affected sinus and reestablishing proper ventilation and drainage [4]. The Caldwell-Luc approach (the canine-fossa approach) to treat FB, a noninvasive fungal contamination, is less and less justified [11]. Nowadays, the pure endoscopic approach should be considered widely the gold standard in treatment of paranasal sinus FB [8, 12], due to its low morbidity and the easy access to the affected paranasal sinus [7].

However, removal of maxillary sinus FB may be long and difficult, in particular when the anterior and/or inferior recesses are involved, as they are notoriously more difficult to manage with the classic endoscopic technique [9, 11].

Therefore, some authors have advocated a combination of the pure endoscopic technique and a complementary endoscopic canine-fossa approach, using a trocar in the canine fossa (the so-called “double approach”) so as to arrive at a complete resection of the fungus ball [13].

As aforementioned, this may make FB surgery in the maxillary sinus with the pure endoscopic technique long and difficult. This leads to an increase in the surgical procedure time, a higher risk of complications due to the difficulty of the technique and, consequently, a higher cost.

Consequently, numerous authors have proposed various techniques, maintaining a pure nasal endoscopic approach (without intervening on the canine fossa). Maxillary sinus areas out of the visual control even with angled scopes (45° and 70° endoscopes) can be assessed endoscopically with a flexible endoscope to assure an overall sinus clearance, as suggested by Pagella et al. [14]. This technique, though effective, necessitates the use of a flexible endoscope dedicated to the operating theatre and is most likely to lengthen the surgical time as the surgical instruments must be changed.

Sawatsubashi et al. have recently proposed an endoscopic technique that combines middle and inferior meatal antrostomies to treat fungal maxillary sinusitis [15]. However, also this proven technique may increase the operating time and, in our opinion, may lead to more comorbidities due to the creation of an unnatural draining passage (i.e., the inferior antrostomy).

Over the last 5 years, our centre has debated the use of a simple and user-friendly technique, proposed for the first time by Chao and Liu in 2006 [9], the so-called “gauze technique.” The merits of this technique do not only lie in its simplicity and the high learning curve but also include a higher speed of execution and lower costs than the technique without gauze, as the materials used are part of the standard supplies in any operating theatre. Moreover, this atraumatic technique has potentially no complications other than those related to the classic endoscopic technique. In order to avoid recurrence, there are two cornerstones, as described by Chao and Liu: to widen the maxillary sinus ostium as much as possible and to take care of pushing the gauze very gently so as to preserve the periosteum of the maxillary sinus; even if the mucosa is injured, it will heal as long as the periosteum is intact [9]. Our personal experience with this technique, respecting these cornerstones, allowed us to obtain a success rate of 96% (24/25 patients) with the gauze technique and a postoperative therapy based only on large-spectrum antibiotics and analgesics without using specific antifungal treatment.

The one relapse treated with a Caldwell-Luc approach may be linked to the fact that this patient had been operated on in emergency for bone damage after a recent elevation of the maxillary sinus floor, presenting with altered maxillary sinus anatomy, and the fungus ball was a chance finding. Indeed, Nicolai et al. show a recurrence rate of 1.4% for maxillary sinusitis (2/135) linked to restenosis of maxillary ostium [4].

In conclusion, although nowadays the treatment of choice for maxillary sinus FB is endoscopic, there are cases when the classic endoscopic technique can make for a difficult removal of pathological material especially if anterior and/or inferior recesses are involved. This study demonstrates how the gauze technique can help avoiding this problem, providing excellent results in the treatment of maxillary FB, using low-cost materials, with a faster execution than the classical endoscopic technique, making for a positive cost/benefit ratio.

## Figures and Tables

**Table 1:** Database.

Patient number	Sex	Age at surgery	Symptoms^*∗*^	Surgical Technique (gauze or no gauze)	Duration of surgery (min)	Nasal unpacking	Hospital days	Postop. events	Follow-up (months)	SNOT20
1	F	68	NC	No gauze	90	48 hours	5	—	105	5
2	F	45	NC/PD/CFP	No gauze	120	24 hours	3	—	98	3
3	F	38	NC/PD	No gauze	90	24 hours	2	—	98	3
4	F	34	PD/CFP	No gauze	180	24 hours	2	Epistaxis (after 5 days)	96	0
5	F	42	NC	Gauze	50	6 hours	4	—	57	4
6	F	57	NC/PD	Gauze	80	24 hours	5	—	53	0
7	M	59	NC/PD	Gauze	150	48 hours	5	—	53	0
8	F	83	NC/PD/CFP	Gauze	50	48 hours	3	—	51	3
9	M	68	NC	Gauze	110	24 hours	4	—	50	0
10	F	31	PD	No gauze	60	24 hours	4	—	48	4
11	F	50	CFP	Gauze	30	24 hours	3	—	48	9
12	F	24	NC/PD/CFP	Gauze (2 times)	80	24 hours	5	—	46	3
13	F	62	NC/PD/CFP	Gauze	60	24 hours	2	—	42	7
14	M	65	NC/CFP	Gauze	50	48 hours	3	—	42	3
15	F	46	NC/PD	Gauze	40	24 hours	3	—	38	35
16	M	61	NC/PD/CFP	Gauze	120	48 hours	3	—	38	5
17	M	48	NC/PD	Gauze	60	24 hours	3	—	35	2
18	F	76	NC/PD	Gauze	70	48 hours	4	—	30	1
19	M	54	NC	Gauze	90	24 hours	2	—	14	5
20	M	41	NC/PD	Gauze (2 times)	50	24 hours	2	—	24	6
21	M	44	NC/PD/CFP	No gauze	90	24 hours	3	—	98	3
22	M	66	NC/PD	Gauze	60	48 hours	4	Relapse (Caldwell-Luc)	31	4
23	M	73	NC/PD	Gauze	50	24 hours	3	—	31	2
24	F	29	NC/CFP	Gauze	120	48 hours	1	—	6	3
25	M	79	PD/CFP	Gauze	70	24 hours	1	—	6	0

^*∗*^NC: nasal congestion, PD: purulent discharge, and CFP: craniofacial pain.

**Table 2:** Statistical analysis.

	No gauze	Gauze	*p* value (non parametric)
Number of subjects	6	19	
Mean age at surgery (SD)	43.3 (13.3)	57.0 (16.2)	0.0697
Mean duration of surgery (SD)	105,0 (41.4)	73.2 (31.8)	0.0466
Mean days of hospitalization (SD)	3.2 (1.2)	3.2 (1.2)	0.9213
Mean SNOT_20 (SD)	3.0 (1.6)	5.0 (7.6)	0.7951
Unpacking timing			0.507
No complication number	5 (83.3%)	19 (100%)	0.240
